# Research on the evolution and the driving forces of land use classification for production, living, and ecological space in China’s Qilian Mountains Nature Reserve from 2000 to 2020

**DOI:** 10.1007/s11356-023-26857-x

**Published:** 2023-04-18

**Authors:** Yaobin Wang, Ruitao Zhao, Ying Li, Rong Yao, Ruoxue Wu, Wenlin Li

**Affiliations:** School of Tourism, Northwest Normal University, Lanzhou, 730070 China

**Keywords:** Nature reserve, Production, living, and ecological space, Functional evolution, Kernel density analysis; Geodetector

## Abstract

With the rapid development of the economy, problems such as resource depletion, environmental degradation, and increasingly strained human-land relations have become increasingly prominent. The rational layout of the production, living, and ecological spaces is the basis for solving the contradiction between economic development and environmental protection. This paper analyzed the spatial distribution pattern and evolution characteristics of the Qilian Mountains Nature Reserve based on the theory of production, living, and ecological space. The results show that the production and living function indexes are rising. The most advantaged areas are in the northern part of the research area, where the terrain is flat and transportation is convenient. The ecological function index rises, falls, then rises again. The high-value area is located in the south of the study area, and its ecological function is intact. The study area is dominated by ecological space. During the study period, the area of production space increased by 858.5 km^2^ and the living space area increased by 341.12 km^2^. The intensification of human activities has separated the continuity of ecological space. The area of ecological space has decreased by 233.68 km^2^. Among geographical factors, altitude has a significant impact on the evolution of living space. Population density is the main socioeconomic factor in changing the areas of production space and ecological space. This study is expected to provide a reference basis for land use planning and sustainable development of resources and environment in nature reserves.

## Introduction

The spatial distribution and functional evolution of the national land pattern are the result of the interaction between human activities and land reconfiguration (Tao et al. 2022; Zhao et al. 2020). Global development and human activities have changed the spatial structure and form of national land, and the functional evolution of spatial patterns is the result of changes in spatial form and structure. Studies have shown (Wang et al. 2018; Yao et al. 2021; Wei et al. 2022) that the unbalanced pattern of agricultural, urban, and ecological spaces reflects the impact of human activities on the natural environment, causing social and ecological problems such as ecological degradation, energy shortages, and natural disasters. The International Geosphere-Biosphere Program (IGBP) and the Human Dimensions in Global Environmental Change Program (IHDP) have proposed the study of land use change patterns and the analysis of driving forces as core research directions to reduce the negative impacts of land use change on nature and society (Qin et al. 2022). From this, land and resource development are shifting from a production space to a model of coordinated production, living, and ecological space (Wang 2021). With socioeconomic development and urbanization, the spatial patterns of production, living, and ecological space clashed, creating a functional contradiction (Zhang et al. 2019; Wang 2022; Wang et al. 2020). Clarifying the production, living, and ecological space has become an important basis for optimizing land resource allocation and building an ecological civilization and further improving the territorial distribution system.

Research on the distribution and function of production, living, and ecological space has gradually become popular in many countries. In the first phase, this paper studies the basic theoretical framework such as the definition of the concept of production, living, and ecological spatial functions, and the identification of features (Luo et al. 2008; Li et al. 2017; Xie et al. 2010; Tang 2015). Conceptual definitions focus on framework systems (Wang et al. 2019), spatial scoping (Brown and Raymond 2014), and the construction of expression paradigms (Zou et al. 2018). There are two streams of research on the identification of functional features. One stream consists of research on the conflict and coordination of production-living-ecological functions within the unit. Scholars explored the pattern of spatiotemporal differentiation in the unit area (Geng et al. 2019) and analyzed their evolutionary characteristics (Wang et al. 2020). The other stream explores the functional differences of regional units, such as the coordinated spatial development of different cities (Wei et al. 2021a) and the regional characteristics of land use (Wei et al. 2021b). The production-living-ecological functions are spatially and temporally heterogeneous and can reinforce, restrict, or eliminate each other. Two aspects of the study are of great significance to regional territorial spatial distribution planning and sustainable development.

The second phase of research focuses on the interrelationship between the production-living-ecological functions and regional coordination, function classification (Zhao et al. 2021a), functional evaluation and optimization (Zhao and Zhao 2023), distribution characteristics (Zhao et al. 2021b), and descriptive methods (Lu et al. 2015). The units of research have been national (Chen et al. 2019), provincial (Li et al. 2021a), river basin (Han et al. 2022), and county (Gao et al. 2022). Scholars have conducted studies on production, living, and ecological space, but there are still some shortcomings. Analysis of spatiotemporal changes and characteristic evolution of the production-living-ecological functions needs to be strengthened. More research has been done on the characteristics of evolution and the pattern of production, living, and ecological space, but the driving factors of their evolutionary pattern evolution have been studied less. Most of the research areas are provincial, urban, and economic circles. Less research has been done on production, living, and ecological space in nature reserves and ecologically vulnerable areas.

The theory of production, living, and ecological space classifies territorial space into production space, living space, and ecological space according to the functional properties of space (Zhao et al. 2019; Yang et al. 2020a; Li et al. 2021b). The three are interrelated and complementary (Liu et al. 2019; Bennett et al. 2009). Production space provides industrial, agricultural, and service products and material security for living space. Living space is the place of human habitation and public activities and allows its inhabitants to survive. The pattern distribution evolved with people’s needs. Ecological space provides the products and services to promote harmonious human-land relations. Economic location advantage and land-intensive use are the main factors of spatial evolution (Chen et al. 2021; Lin et al. 2020). Tourism market demand and policy orientation can explain the evolution of territorial spatial patterns and functions in industrial regions dominated by tourism (Xi et al. 2014; Chen and Jin 2015). Production, living, and ecological space are driven by external factors that change the distribution of space and of production-living-ecological functions. The transformation of spatial functions is the main process of spatial production, but also the result of the synergistic development of humans and nature. The production, living, and ecological space theory can explain the relationship between human activities and the natural environment more fully than the human-earth relationship theory (Zhou et al. 2020a).

Taking China’s Qilian Mountains Nature Reserve as an example, the period 2000–2020 is chosen as the research period. According to this research needs, 30 m of raster land cover remote sensing monitoring data of this area in 2000, 2005, 2010, 2015, and 2020 were selected and reclassified according to the theories of production, living, and ecological space. This paper analyzes the distribution and evolutionary characteristics of production, living, and ecological space, explores the spatial variation characteristics of land function conversion, and reveals the pattern and evolution of production, living, and ecological space in the Qilian Mountains. The geodetector was used to analyze possible factors affecting the evolution of regional production, living, and ecological patterns. The purpose of this paper is to evaluate the evolution characteristics of the production, living, and ecological functions in the Qilian Mountains Nature Reserve, clarify the spatiotemporal variation of the production, living, and ecological space area, and explore the driving factors behind it. This paper expects to provide suggestions for land use planning and sustainable development of resources and the environment in the same type of nature reserves.

## Research methodology and data sources

### Study area overview

**Fig. 1:**
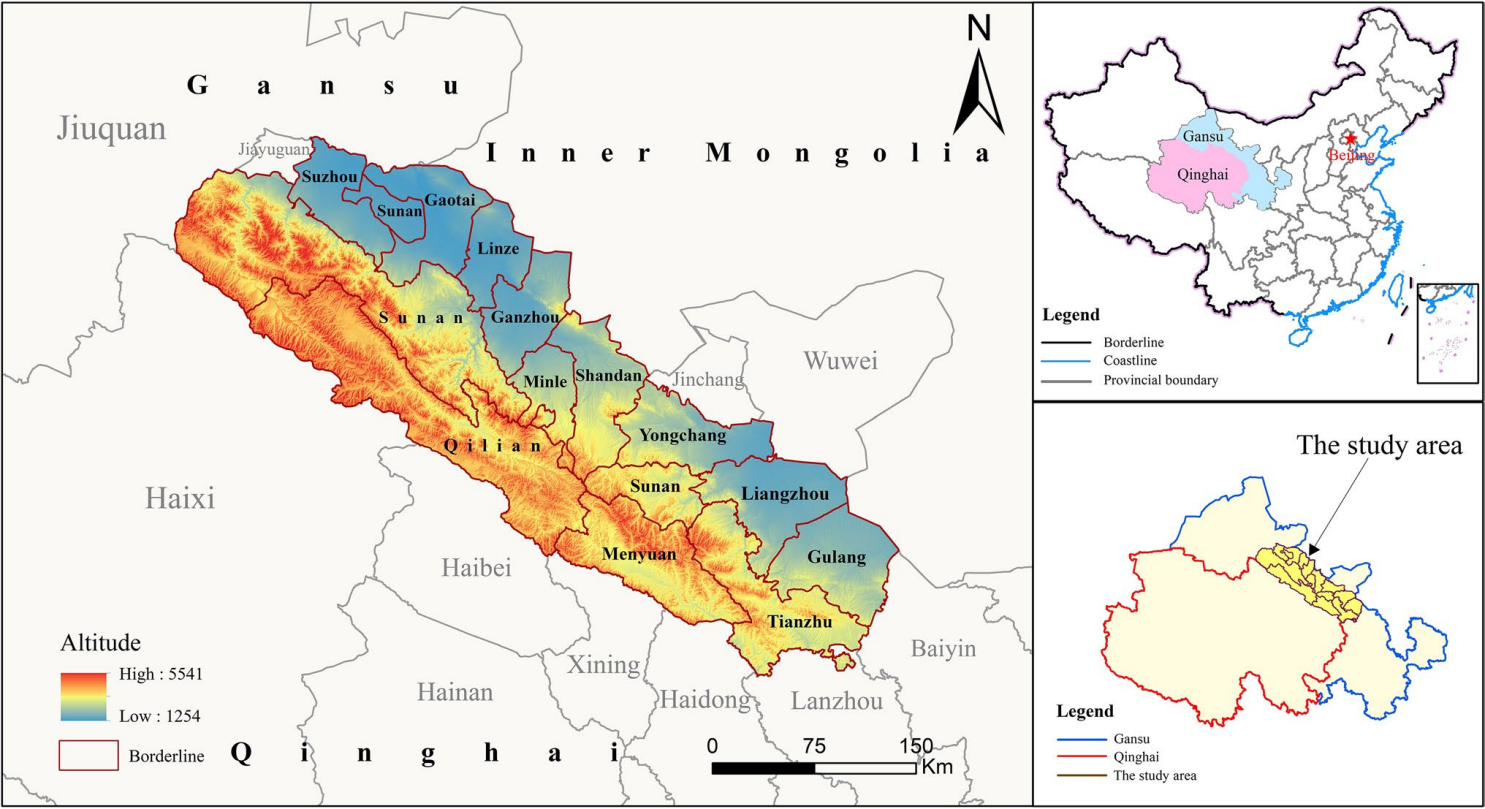
Location map of Qilian Mountains Nature Reserve

China’s Qilian Mountain Nature Reserve (Fig. 1) is located at the border of Qinghai and Gansu provinces on the northern edge of the Qinghai-Tibet Plateau, at an altitude of 1684 ~ 5604 m, with geographical coordinates of 94°10'–103°04'E, 35°50'–39°19'N. The Qinghai-Tibet Plateau in the south and the Hexi Corridor in the north, with diverse natural ecosystems and abundant wildlife resources, are extremely important glaciers, water conservancy ecological function areas, wildlife migration corridors, biological resource banks, and genetic banks in China, as well as important ecological function areas and safety barriers. The reserve is located in the continental climate zone of the plateau, with strong solar radiation, large temperature difference between day and night, long cold season, short warm season, distinct dry and wet seasons, rain, and heat in the same season. The average annual temperature is below 4 ℃, the maximum extreme temperature is 37.6 ℃, and the minimum extreme temperature is − 35.8 ℃. The average annual precipitation is 400 mm. The protected areas range from northwest to southeast, with a narrow strip of land covering a total area of 5.02 × 104 km^2^ and involving eight nature reserves, with 27,500 km^2^ of core reserves and 22,700 km^2^ of general control areas. With rapid economic and social development, the development and construction of the region have intensified, production and living space have begun to expand, and the contradiction between land supply and demand has become increasingly tense (Pan et al. 2022). Aligning production-living-ecological with economic development, resolving the contradiction of land supply and demand, and optimizing the land use structure have become urgent problems in the Qilian Mountain Nature Reserve.

### Data source

This research relies on remote sensing data and socioeconomic development data. The land use remote sensing data were obtained from the Resource and Environment Science Data Center of the Chinese Academy of Sciences (http://www.resdc.cn). Landsat TM/OLI remote sensing images with cloud coverage < 10% from June to September were selected with a spatial resolution of 30 m and kappa coefficients, all greater than 0.88. Five periods of current land use data were generated for 2000, 2005, 2010, 2015, and 2020. Average elevation and average slope factors were obtained from geospatial data from cloud DEM. Based on the land use classification system and relevant studies (Xie et al. 2021; Deng and Yang 2021), land use space types are divided into four categories: production space, living space, ecological space, and unused land. Socioeconomic development data come from county and municipal statistical yearbooks, statistical bulletins and the “Gansu Statistical Yearbook,” the “Qinghai Statistical Yearbook,” and the “China County Statistical Yearbook.” Data for Ganzhou District were not available around 2000 and were supplemented by data for 2001. The outbreak of COVID-19 in 2020 had a devastating impact on tourism, so total tourism revenue for 2020 was replaced with data from 2019. Some counties and cities with missing data were supplemented by linear interpolation (Table 1).

**Table 1:** Land use classification and type

Land use classification	LUCC remote sensing monitoring data classification
Production space	11 paddy field, 12 dry land
Living space	51 town, 52 rural settlement, 53 construction land
Ecological space	21 forested, 22 shrub land, 23 open forest land, 24 other forest land, 31 high-cover grassland, 32 medium-cover grassland, 33 low-cover grassland, 41 rivers and canals, 42 lakes, 43 reservoir ponds, 44 permanent glacial snow, 45 mudflats, 46 bench
Unused land	61 sandy land, 62 gobi, 63 saline land, 64 marshland, 65 bare land, 66 bare rocky land, 67 other

## Research methodology

### Entropy method

The entropy method is an objective assignment method, which can avoid the subjective influence in weight acquisition. Based on cross-sectional data, the entropy value method is used to determine the weight of the indicators of regional production-living-ecological functions. The linear weighting method is used to calculate the comprehensive functions score of counties and cities in the Qilian Mountains area for the corresponding years and to make the corresponding image. The steps are as follows (Gong et al. 2022; Li et al. 2020).

1. Set the indicators. There are *m* counties and cities, *n* evaluation indicators, and *T* years, which together constitute the initial global evaluation matrix.
  1 $$X={\left\{{x}_{ij}^{t}\right\}}_{{m}_{T\times n}}$$

In formula (1), ${x}_{\mathrm{i}j}^{t}$ represents the *j-*th index value of the *i*-th counties and cities in year *t*.

(2) Process the data. To avoid the interference of dimensionality and positive and negative orientations among the indicators, the data matrix was standardized dimensionless using polarization normalization. To eliminate the effect of negative and zero on the results of the algorithm, the data were shifted and the positive indicators were normalized using formula (2):
  2 $${{y}^{t}}_{i\dot{J}}=\frac{{x}_{ij}^{t}-{x}_{j\mathrm{min}}}{{x}_{j\mathrm{max}-}-{x}_{j\mathrm{min}}}+0.01$$

In formula (2), *i* = 1,2, …, *m*; *j* = 1,2, …,* n*; *t* = 1,2, …, *T*.

Formula (3) is used for negative indicators:

3 $${{y}^{t}}_{i\dot{J}}=\frac{{x}_{j\mathrm{max}-}{x}_{ij}^{t}}{{x}_{j\mathrm{max}-}-{x}_{j\mathrm{min}}}+0.01$$

In formula (3), *i* = 1,2, …, *m*; *j* = 1,2, …, *n*; *t* = 1,2, …,* T*.

(3) Calculate the entropy value.
  4 $${e}_{j}=-k\sum\nolimits_{t=1}^{T}\sum\nolimits_{i=1}^{m}{{p}^{t}}_{ij}\mathrm{ln}{p}_{ij}^{t}$$

In formula (4), *e*_*j*_ is the entropy value of the *j-*th index. $p_{ij}^t=y_{ij}^{t'}$/$\sum_{t=1}^{T}\sum_{i=1}^{m}{{y}^{t}}_{ij}$, ${p}_{ij}^{t}$ is the share of the *i*-th indicator value in year *t* under the* j*-th indicator.* k* = 1/ln(*mT*),* k* is determined by the number of counties and cities and the number of years.

(4) Determine weights.
  5 $${w}_{j}=(1-{e}_{j})/\sum\nolimits_{j=1}^{n}(1-{e}_{j})$$

In formula (5), ${w}_{j}$ is the weight of the *j*-th indicator. 0 ≤ ${w}_{j}$  ≤ 1, $\sum_{j=1}^{n}{w}_{j}=1$. The details are shown in Table 2.

(5) Calculate the production-living-ecological functions comprehensive evaluation score.
  6 $${S}_{j}=\sum\nolimits_{j=1}^{n}{w}_{j}{{y}^{t}}_{i\dot{J}}$$

**Table 2:** Functional evaluation indexes and weights of the production-living-ecological functions in the Qilian Mountains area

Land use classification	Indicator	Indicator interpretation or calculation method	Weights
Production functions	Arable land per capita ( +)	Total arable land/village population (hm^2^/pp)	0.198
	Grain production per capita ( +)	Total food production/number of rural population(kg/pp)	0.189
	Agricultural output value per capita ( +)	Total agricultural output value/number of village population(yuan/pp)	0.291
	Agricultural modernization level ( +)	Total power of agricultural machinery/area of cultivated land (kw/hm^2^)	0.184
	Share of non-agricultural employment ( +)	Rural non-farm employment/number of rural employees (%)	0.138
Living functions	Net income per rural resident ( +)	Reflecting the average income level of rural residents (yuan)	0.278
	Rural Engel coefficient ( −)	Rural residents' expenditure on food consumption/total household expenditure (%)	0.191
	Rural housing area per capita ( +)	Reflecting the housing level of rural residents (m^2^/pp)	0.125
	Annual electricity consumption per capita in rural areas ( +)	Rural electricity consumption/rural population (kw-h/pp)	0.198
	Number of medical beds per 10,000 population ( +)	Total number of beds in hospitals and health centers/rural population (beds/million people)	0.208
Ecological functions	Fertilizer input intensity ( −)	Application of agricultural fertilizer/total arable land area (kg/hm^2^)	0.171
	Rural water resources per capita ( +)	Total water resources/total rural population (m^3^/pp)	0.308
	Forest coverage rate ( +)	Total area of forest land/total land area (%)	0.226
	Grassland coverage rate ( +)	Total area of grassland/total land area (%)	0.108
	Amount of agricultural plastic film used per unit area ( −)	Amount of agricultural plastic film used/total arable land area (kg/hm^2^)	0.187

“pp” means per person

In formula (6), ${s}_{j}$ is the composite score of the production-living-ecological functions, and ${w}_{j}$ is the weight of the index obtained.

### Land use transfer matrix

The land use transfer matrix is a method to research the land use status and the different types of land use circulation and to reflect the structural characteristics of land use change (Mikelsone et al. 2021). The spatial analysis function of ArcGIS10.8 can summarize the spatial transfer matrix of the production, living, and ecological space from 2000 to 2005, 2005 to 2010, 2010 to 2015, and 2015 to 2020. This is the most accurate way to analyze the transformation of the functional structure of land use in China’s Qilian Mountains Nature Reserve. The mathematical expression is

7 $${S}_{ij}=\left[\begin{array}{cc}\begin{array}{ccc}{S}_{11}& {S}_{12}& \cdot \cdot \cdot \\ {S}_{21}& {S}_{22}& \cdot \cdot \cdot \\ \cdot \cdot \cdot & \cdot \cdot \cdot & \cdot \cdot \cdot \end{array}& \begin{array}{c}{S}_{1n}\\ {S}_{2n}\\ \cdot \cdot \cdot \end{array}\\ \begin{array}{ccc}{S}_{n1}& {S}_{n2}& \cdot \cdot \cdot \end{array}& {S}_{nn}\end{array}\right]$$

In formula (7), ${S}_{ij}$ is the area km^2^ of land use type *i* transformed to *j*, *i* and* j* denote the land use types at the beginning and end of a research period, *S* denotes the land use area, and *n* denotes the number of land use types before and after land transfer.

### Land use type dynamic attitude

The land use type dynamic attitude describes the rate of change of various types of land use over a certain period and reflects the result of the change of types of land use area, which is conducive to exploring the evolution patterns and driving factors of land use (Xiao et al. 2022).

8 $$K=\frac{{u}_{b}-{u}_{a}}{{u}_{a}}\times \frac{1}{T}\times 100\mathrm{\%}$$

In formula (8),* K* denotes the dynamic attitude of a land use type during the study period, and *T* is the research period. *U*_*a*_, *U*_*b*_ denote the area of that land use type at the beginning and end of the study period.

### Kernel density estimation

Kernel density estimation is a non-parametric test based on unknown density function estimation, which can intuitively reflect the distribution probability (François-Rémi and Pierre-Yves 2022). In this paper, the kernel density estimation method is used to analyze the spatial distribution characteristics of the transformed area of production, living, and ecological space. Convert different functional patch elements into point elements, calculate the data clustering in the region, and create point peaks or kernels to create smooth and continuous surfaces with density values indicating the distribution of sample points within the study area. Estimating the kernel density can help to understand the location distribution characteristics of the area transformation of the production, living, and ecological space in China’s Qilian Mountains area and provide a scientific basis for land space optimization. The kernel density estimation is calculated as follows.

9 $${F}_{n}\left(x\right)=\frac{1}{nh}\sum\nolimits_{i=1}^{n}k\left(\frac{x-{x}_{i}}{h}\right)$$

In formula (9), ${F}_{n}\left(x\right)$ is the kernel density estimate of the production, living, and ecological space transitions to and from the surface. *h* is the search radius (m). *k* is the kernel density function. *n* is the number of samples of the production, living, and ecological space transitions to and from the centroid. $x-{x}_{i}$ is the estimated distance between two centroids (m).

### Geodetector

Geodetector is a statistical method for detecting spatial differentiation and revealing the driving forces behind them. It is not subject to excessive conditions (Wang and Xu 2017) and overcomes the shortcomings of traditional statistical methods for dealing with variables. Geodetector is used to quantitatively assess the factors affecting the production-living-ecological functions of the research area and the intensity of their influence; factor detection is used to detect the spatial heterogeneity of Y and the degree to which a certain factor X explains the spatial heterogeneity of Y. Combining the characteristics of the study area and data accessibility, the research selects the production-living-ecological functional drivers from both natural and socioeconomic and uses factor detection and interaction detection to study the spatial pattern changes of the production-living-ecological functions in China’s Qilian Mountains area and the interaction among the drivers. The formula for differentiation and factor detection is below:

10 $${q}_{D}=1-\frac{1}{N{\sigma }^{2}}\sum\nolimits_{i=1}^{m}{N}_{Di}{{\sigma }_{i}}^{2}$$

In formula (10), ${q}_{D}$ is the degree of influence of driver *D* on the driving changes in the spatial pattern of the three lives, ${q}_{D}\in$[0,1]; ${q}_{D}$ = 0 means that the driver is completely unrelated to the spatial change of the production, living, and ecological space, and the larger the value of ${q}_{D}$, the stronger the driver’s ability to explain the change in the spatial pattern of the production, living, and ecological space, and the weaker the opposite. ${q}_{D}$ = 1 indicates that the driver fully controls the spatial pattern change of the production, living, and ecological space. *n* is the number of samples in the study area. ${{\sigma }_{i}}^{2}$ is the variance of the spatial rate of change of the three lives in the region. *i* represents the group category for driver *D*.* m* is the total number of partitions for driver *D*.

Interaction is detected by comparing the influence of each factor on the variable independently or after interaction and determining the strength of the influence. There are five kinds of interaction between two factors: nonlinear attenuation, two-factor enhancement, single-factor nonlinear attenuation, nonlinear enhancement, and mutual independence.

## Results and analysis

### Analysis of the spatial distribution pattern and evolution characteristics

According to the evaluation system of production-living-ecological functions in China’s Qilian Mountains area, the synthetic score is calculated by ArcGIS10.8 natural breakpoint method. The results of production-living-ecological functions are divided into four regional categories: low-value, medium–low value, medium–high value, and high-value. The distribution and evolution characteristics of each are analyzed.

**Fig. 2:**
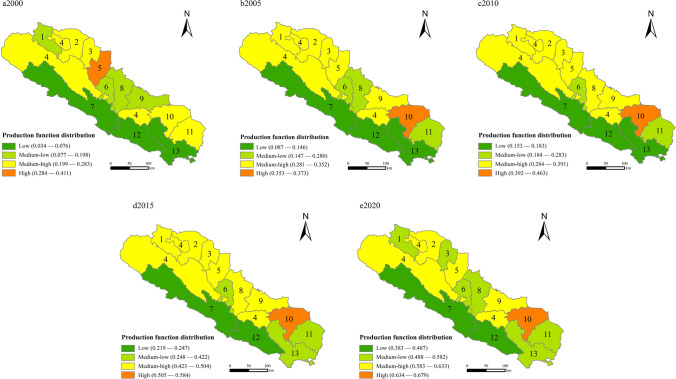
Distribution of production functions in the Qilian Mountains area in 2000–2020

Production functions: From 2000 to 2015, China’s Qilian Mountains area showed a year-on-year increase in the composite index score of production function (Fig. 2). COVID-19 affected the production and service functions of Linze and Shandan in the north in 2020. The development trend in the north was better than those in the east and south, and the production and functions in Gansu were better than in Qinghai. High-value production function areas and medium–high-value areas were distributed in Liangzhou, Yongchang, Shandan, and Minle. The flat terrain and convenient transportation contribute to better economic production conditions, attracting technology, talent, and capital. Industry agglomeration capacity laid a good foundation for ecotourism.

Low and medium–low-value areas were concentrated in the eastern and southern Qilian Mountains region. More than 80% of the land in the region was hilly and mountainous with closed transportation, low economic density, limited industrial development conditions, and a weak production service infrastructure. High-value areas should spread production functions outward, optimize industrial structure and transportation capacity, and build up underdeveloped areas such as Menyuan and Qilian. At the same time, the management should introduce policies to develop ecotourism and other green industries to protect ecological sustainability. (Remarks: 1. Suzhou; 2. Gaotai; 3. Linze; 4. Sunan; 5. Ganzhou; 6. Minle; 7. Qilian; 8. Shandan; 9. Yongchang; 10. Liangzhou; 11. Gulang; 12. Menyuan; 13. Tianzhu.)

**Fig. 3:**
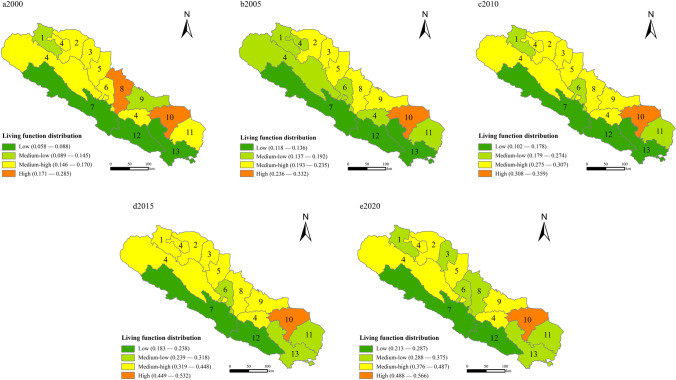
Distribution of living functions in the Qilian Mountains area in 2000–2020

Living functions: From 2000 to 2020, there was a clear distribution of living functions in the Qilian Mountains area (Fig. 3). The comprehensive value gradually increased, and there was considerable overlap with the distribution of production functions. From 2000 to 2005, there was a slight decline in the living functions of Sunan and Minle. With better commercial services and social security conditions, the standard of living in the Qilian Mountains area has risen. From 2010 to 2015, the standard of living in the Qilian Mountains area tended to be favorable. COVID-19 caused some decline in living standards.

The low and middle-low-value areas are in Menyuan and Qilian in Qinghai, Gulang, and Tianzhu in Gansu. These areas were located at the eastern end of the Qilian Mountains and the southern end of the Daban Mountains, with mountainous hills, complex topography, vast differences in altitude height, substandard living conditions, poor quality of arable land, and weak infrastructure and services. Since rapid economic development has led to a severe population decline in rural areas, regional development should be coordinated to promote adequate and habitable living space.

**Fig. 4:**
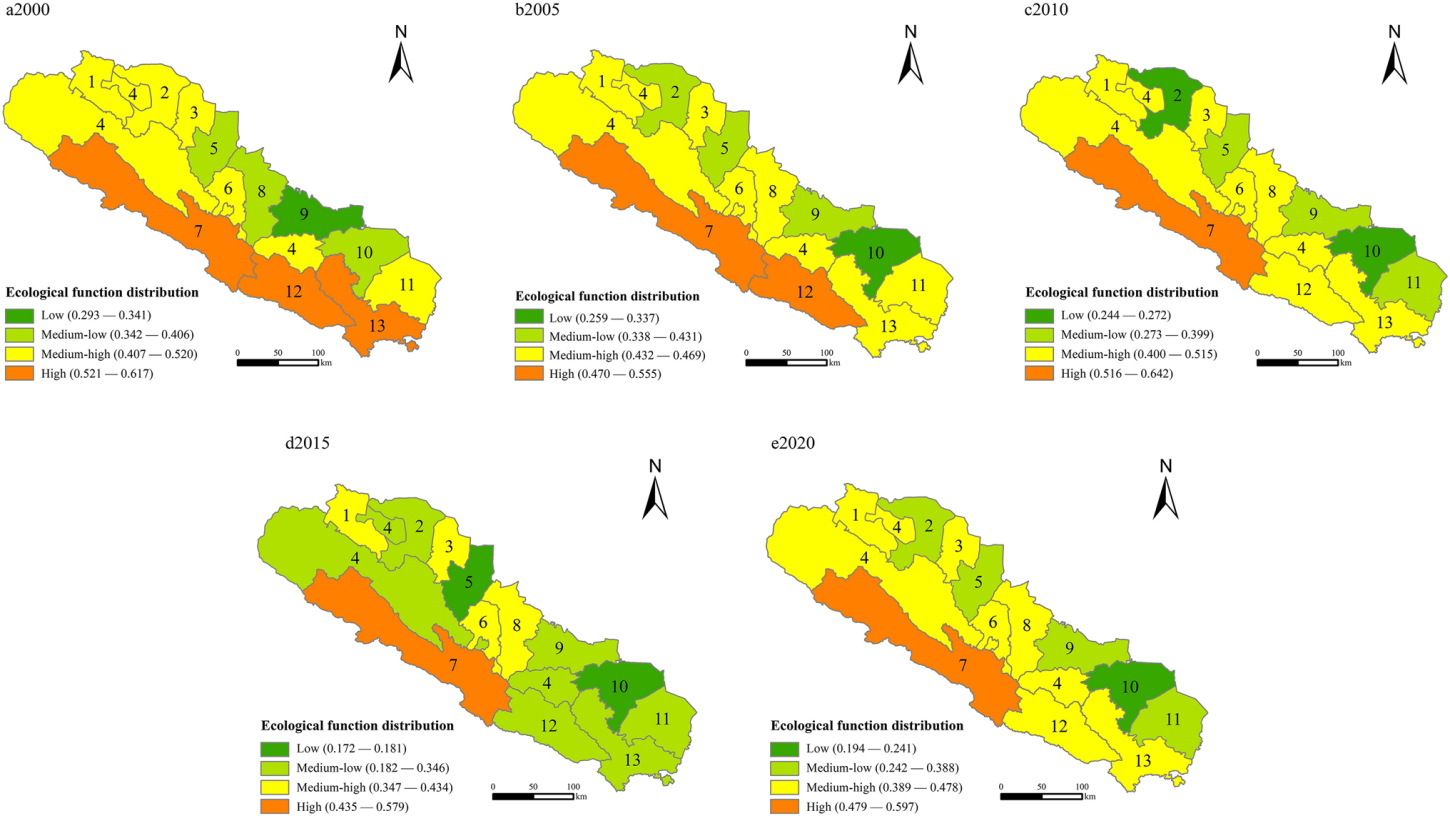
Distribution of ecological functions in the Qilian Mountains area in 2000–2020

Ecological functions: The pattern of ecological functions change in the Qilian Mountains area was obvious from 2000 to 2020 (Fig. 4). The high and medium–high-value areas were located in Menyuan and Qilian in Qinghai, where ecological functions were intact and biodiversity was concentrated. Low and medium–low-value areas were distributed in northern Liangzhou and Gaotai. These areas were densely populated industrial clusters. Large-scale economic production and construction have a great influence on the ecological environment, and the ecological service functions are poor.

From 2000 to 2010, the value of ecological functions showed an upward trend. Between 2010 and 2015, the ecological service functions in the research area were significantly weakened, ecological service functions in the areas of Sunan, Menyuan, and Tianzhu deteriorated, and ecological damage was severe. Large-scale mining, illegal construction of hydropower facilities, and reckless ecotourism at the expense of resource protection brought environmental problems to the fore. In 2017, the state enacted protection policies and launched special measures to improve the environment. The ecological functions of the Mengyuan and Sunan areas were alleviated. The Qilian Mountains area, as a fragile ecosystem, should take advantage of the ecological functions of the south to construct ecological barriers and clear the ecological space.

### Changes in the production, living, and ecological space

**Fig. 5:**
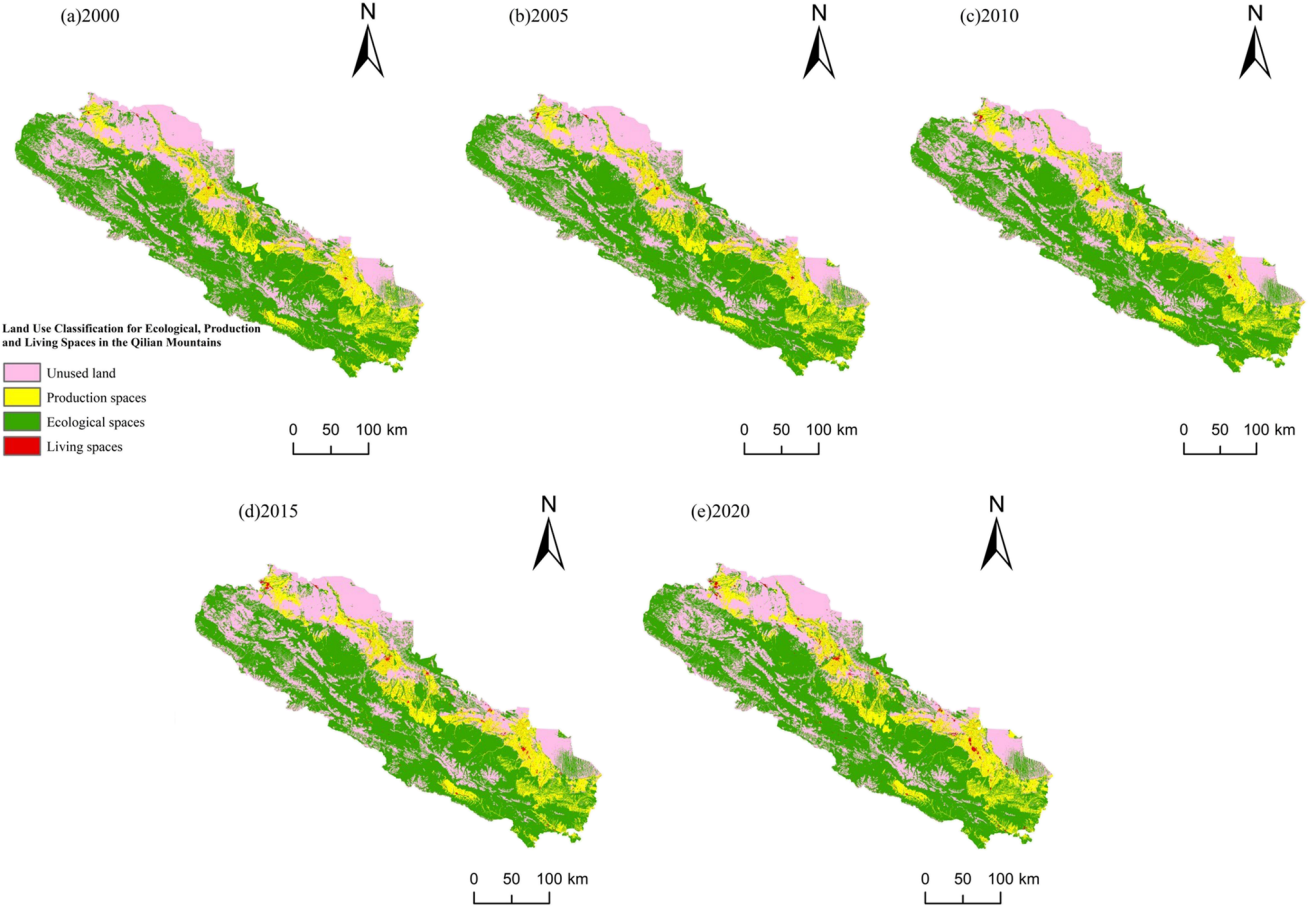
Area change of land use function type in the Qilian Mountains area from 2000 to 2020

Figure 5 showed that among the production, living, and ecological space in the Qilian Mountains area in five periods, the largest ecological functional space was the south-central region, which covered the central and southern regions. The second was the unused land, which was distributed in the south-central ecological block northwest of the Qilian Mountains. The living space was the smallest, dotting the eastern and northern Qilian Mountains. The trend of spatial change in land use function from 2000 to 2020 was as follows. Production and living space continued to increase, but ecological space and unused land decreased.

According to Table 2, with population growth and social and economic development in the counties surrounding the Qilian Mountains, demand for and supply of agricultural production and urban living space have grown. In 2020, the living space was 1029.48 km^2^, nearly twice what it had been in 2000, and the production space has increased to 858.5 km^2^. From 2000 to 2010, both increased significantly and the area of unused land shrank, indicating the accelerated reclamation and development of unused land. Between 2000 and 2020, the area of unused land decreased by 1068.76 km^2^ as production and living space increased, indicating that the changes in production and living space in the research area have a greater impact on unused land. The increase or decrease in the area remained consistent over the four periods, indicating some correlations.

Although the Qilian Mountains area has the smallest proportion of living space, problems have arisen with urbanization and the development of ecotourism. The increase in demand for production and living space, the blind pursuit of economic benefits generated by ecotourism, and the neglect of ecological, environmental protection have led to a reduction in the area of ecological space. This area should protect the ecological environment while planning the layout of production and living space, reclaiming unused land, improving soil erosion, and laying the foundation for sustainable regional economic and ecological development (Table 3).

**Table 3:** Land use in Qilian Mountains, 2000–2020 (km^2^)

Land use classification	Area					Increase or decrease changes				
	2000	2005	2010	2015	2020	2000—2005	2005—2010	2010—2015	2015—2020	2000—2020
Production space	10,987.69	11,406.59	11,639.61	11,721.33	11,846.19	418.9	233.02	81.72	124.86	858.5
Living space	688.36	721.98	792.08	883.23	1029.48	33.62	70.1	91.15	146.25	341.12
Ecological space	44,929.21	44,795.56	44,674.76	44,642.45	44,695.53	− 133.65	− 120.8	− 32.31	53.08	− 233.68
Unused land	26,628.32	26,309.47	26,089.09	25,986.38	25,559.56	− 318.85	− 220.38	− 102.71	− 426.82	− 1,068.76

### Analysis of the evolution of land use function in the Qilian Mountains area

**Fig. 6:**
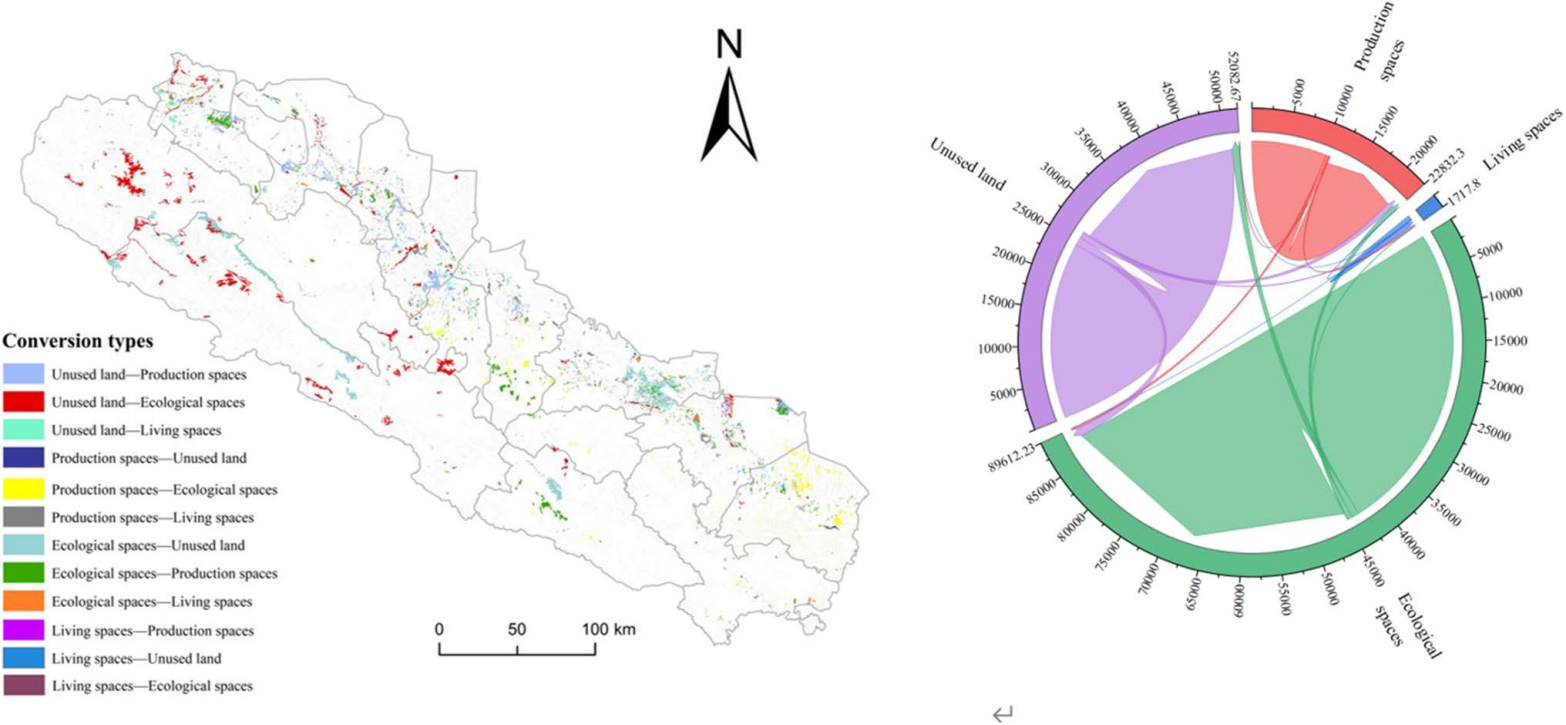
Transformation characteristics of production, living, and ecological space in Qilian Mountains from 2000 to 2020

To explore the conversion model of land use function type in the Qilian Mountains area, the land use transformation matrix from 2000 to 2005, 2005 to 2010, 2010 to 2015, 2015 to 2020, and 2000 to 2020 was obtained (Table 4), and the distribution for 2000–2020 was visualized (Fig. 6).

**Table 4:** The transfer matrix of land use function in the Qilian Mountains area (km^**2**^)

2000–2005							2005–2010				
	Production space	Living space		Ecological space	Unused land		Production space	Living space	Ecological space		Unused land
Production space	10,886.49	1.21		259.32	259.53		10,847.59	31.24	431.19		329.56
Living space	12.91	686.46		4.59	18.02		49.09	687.32	20.47		35.19
Ecological space	72.88	0.44		44,598.68	123.52		420.19	2.11	43,204.51		1057.17
Unused land	15.39	0.27		66.56	26,227.23		89.52	1.31	1138.36		24,886.56
2010–2015							2015–2020				
	Production space	Living space		Ecological space	Unused land		Production space	Living space	Ecological space		Unused land
Production space	11,468.54	15.34		123.28	113.96		11,127.15	59.69	404.03		254.55
Living space	43.91	774.05		16.87	48.40		81.19	781.42	52.24		114.61
Ecological space	102.30	1.73		44,396.60	140.34		330.81	21.16	43,542.29		798.41
Unused land	24.63	0.95		146.75	25,812.12		181.48	20.94	636.49		24,718.32
2000–2020											
	Production space		Living space			Ecological space				Unused land	
Production space	10,407.45		52.98			670.05				714.93	
Living space	146.14		625.43			73.55				184.33	
Ecological space	363.80		7.73			43,172.59				1147.43	
Unused land	69.50		2.21			1004.49				24,479.89

The results of the analysis showed that the transfer areas of production and living space from 2000 to 2020 were 1437.96 km^2^ and 404.02 km^2^, respectively. The increase in production space was concentrated in the northern (Ganzhou, Linze, Shandan) and northwestern (Suchuan, Gaotai) parts of the research area and came mainly from the transfer of ecological space and unused land. With the rapid development of agriculture and industry, production space continues to occupy ecological space, causing pollution and other problems. The highest conversion rates were recorded in 2005–2010 and 2015–2020, with conversion areas of 431.19 km^2^ and 404.03 km^2^, respectively.

During production, the regions of Gao Tai, Linze, and Ganzhou have converted unused desert, saline land, and wasteland into productive economic spaces, increased the area of cultivated land, solved problems such as soil erosion, and set an example for other regions. The increased living space comes from 146.14 km^2^ of production space and 184.33 km^2^ of unused land in central Liangzhou and southeastern Yongchang. With urbanization, living space has consumed production resources, but there is also unused land. A total of 1004.49 km^2^ of ecological space has been transformed into unused land in the northern part of Qilian County and the central part of Menyuan County in Qinghai Province. Grassland degradation, lake shrinkage, and river decline have limited the possibility of ecologically sustainable development. It is necessary to strengthen the ecological restoration and emergency protection of ecological functions, establish an ecological, environmental monitoring network, improve the ecological governance system, and improve ecological protection capabilities. About 1147.43km^2^ in the southwest of the study area has been transformed into an ecological space which provides references for ecologically degradation area through the closure of mountains for forest cultivation, strengthening of comprehensive management and the implementation of green projects such as ecotourism.

In terms of types of land transfers (Table 5), the area of production space changed the most among the production, living, and ecological space (49.56%) and showed a year-on-year increase, with the fastest increase in 2015–2020 (16.56%). The change in ecological space was − 0.52%, with only positive changes between 2015 and 2020. In terms of land use dynamics (Table 6), the production space grew steadily and the dynamic share of living space increased, peaking at 3.31% in 2015–2020.

**Table 5:** 2000–2020 the transfer-in and transfer-out area of production-living-ecological functions in the Qilian Mountains area (km^2^)

2000–2005							2005–2010				
	Transfer in	Transfer out		Amount of change	Magnitude of change		Transfer in	Transfer out	Amount of change		Magnitude of change
Production space	35.52	1.91		33.61	3.81%		104.76	34.65	70.11		2.04%
Living space	520.05	101.18		418.87	4.88%		792.00	558.80	233.20		9.71%
Ecological space	196.84	330.48		− 133.64	− 0.30%		1479.46	1590.03	− 110.57		− 0.27%
Unused land	82.23	401.07		− 318.84	− 1.2%		1229.19	1421.93	− 192.74		− 0.84%
2010–2015							2015–2020				
	Transfer in	Transfer out		Amount of change	Magnitude of change		Transfer in	Transfer out	Amount of change		Magnitude of change
Production space	109.17	18.02		− 61.66	0.70%		248.03	101.79	146.24		1.07%
Living space	252.58	170.83		81.75	11.51%		718.27	593.48	124.79		16.56%
Ecological space	250.77	286.9		− 36.13	− 0.07%		1150.38	1092.77	57.61		0.12%
Unused land	172.33	302.7		− 130.37	− 0.39%		838.92	1167.56	− 328.64		− 1.64%
2000–2020											
	Transfer in		Transfer out			Amount of change				Magnitude of change	
Production space	404.02		62.92			341.1				7.81%	
Living space	1437.96		579.44			858.52				49.56%	
Ecological space	1518.96		1748.09			− 229.13				− 0.52%	
Unused land	1076.2		2046.69			− 970.49				− 4.01%

**Table 6:** Dynamic attitude to land use

Land use dynamic attitude					
Land use classification	2000–2005	2005–2010	2010–2015	2015–2020	2000–2020
Production space	0.76%	0.41%	0.14%	0.21%	0.39%
Living space	0.98%	1.94%	2.30%	3.31%	2.48%
Ecological space	− 0.06%	− 0.05%	− 0.01%	0.02%	− 0.03%
Unused land	− 0.24%	− 0.17%	− 0.08%	− 0.33%	− 0.20%

From 2000 to 2005, the change in land use in ecological space showed a negative trend, indicating that human activities squeezed ecological space, and over-exploitation of natural resources caused pollution. From 2015 to 2020, the dynamics of land use increased, consistent with the trend of the magnitude of change, indicating that the local governments recognized the contradiction between human and ecological space and formulated conservation policies to resolve that contradiction. From 2000 to 2020, the dynamics of unused land were negative, indicating that with population growth, unused land was developed, which alleviated the land use problem of production, living, and ecological space.

### Spatial differentiation characteristics

**Fig. 7:**
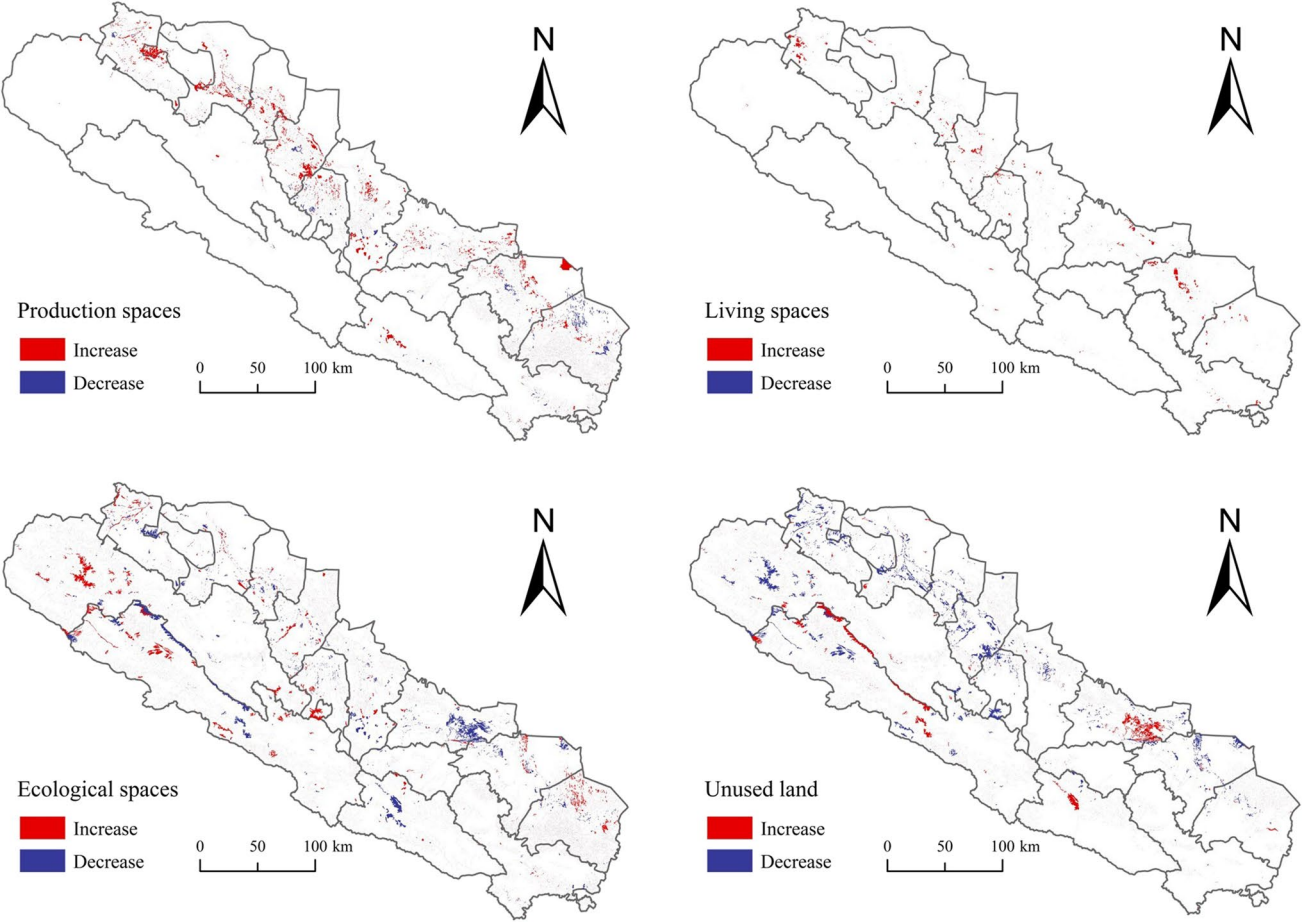
Changes in the production, living, and ecological space from 2000 to 2020

To study the functional transformation characteristics of production, living, and ecological space in the Qilian Mountains area and to analyze the spatial distribution pattern, the production space patches transferred in were defined as patches increasing in 2020 compared with 2000, and the production space patches transferred out were defined as patches decreasing in 2020 compared with 2000. The other three spatial types were defined in the same way and mapped in ArcGIS (Fig. 7).

The evolution of production, living, and ecological space in the Qilian Mountains area is uneven. Production space increased significantly, concentrated in Suzhou, Gaotai, Linze, and Ganzhou in Gansu Province, while Mengyuan and Qilian in Qinghai Province have not noticeably changed. The living space did not change significantly, but there has been a small increase in central Liangzhou and western Suzhou. The decrease in ecological space was more obvious, concentrated in the south-central part of Yongchang and the northern part of Qilian-Sunan overlap, with a significant increase in central Suzhou.

The decrease in unused land was concentrated in the western and northern parts of the research area, while growth was observed in northern and southern Qilian, Subei, and central Yongchang. It was consistent with the decrease in ecological space in the region, indicating that desertification in the region was due to unfavorable ecological protection systems and that the implementation of institutional policies and rational use of mountain resources have become particularly important.

**Fig. 8:**
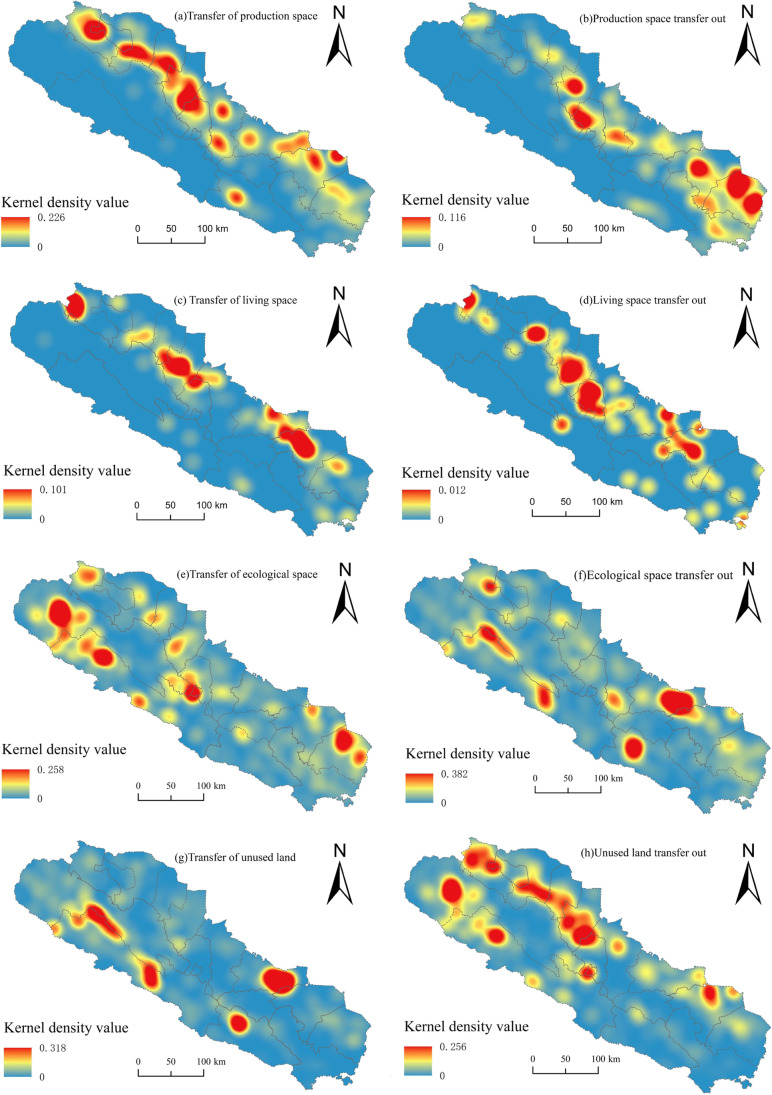
Spatial characteristics of kernel density in the Qilian Mountains area for the conversion of the production, living, and ecological space from 2000 to 2020

To study the spatial variations of the functional transition in the Qilian Mountains area, kernel density analysis was introduced. The density and scale of the conversion of production, living, and ecological space were researched by changing the number of patches to points in Fig. 7, and the spatial differentiation characteristics were analyzed, as shown in Fig. 8.

It can be seen from Fig. 8a,b that the area of production space transferred in 2000–2020 showed obvious spatial clustering characteristics, and the kernel density values transferred in were significantly larger than those that were transferred out. The high-value areas were mainly distributed in Su Zhou, Gaotai, Linze, and Ganzhou.

The spatial distribution concentration of production space was less concentrated than increases, and the high-value areas were distributed in Gulang and Liangzhou. The spatial clustering of the increase in living space was high, and the areas with high kernel density values were distributed in Yongchang, Liangzhou, and Ganzhou, with a kernel density value of 0.101. The kernel density transferred out from living space to other spaces was low, with a density maximum of only 0.012. The decrease of ecological space was the most obvious feature of production, living, and ecological space evolution in the Qilian Mountains area (Fig. 8f). The spatial clustering of ecological space reduction was very high, and its high kernel density area was located in Yongchang and Menyuan, with values as 0.382.

The high-value areas of ecological space transfer were widely distributed, the spatial agglomeration was low, the uniformity was high, and their kernel density value was 0.258. The result shows that the area converted from ecological conservation space to other spaces in the unit was larger than the area transferred from other functions to ecological conservation space.

From 2000 to 2020, the area of ecological space in the Qilian Mountains area declined, consistent with previous research. Figure 8g,h showed that the spatial clustering of the reduced area of unused land in the Qilian Mountains area was higher than the increased area of unused land. The decrease in the area of unused land was in Suzhou, Gaotai, Linze, and Ganzhou, which were areas with a greater increase in production space, and consistent with the previous analysis that the spatial area of unused land in the Qilian Mountains area was decreasing. The high-value area of unused land increase was located in Yongchang, Menyuan, and other regions with high spatial concentration and the highest value of kernel density was 0.318.

### The driving forces of divergences in the Qilian Mountains area

#### Analysis of impact factors

The evolution of the spatial pattern was influenced by the combination of geographical and socioeconomic factors. Geographical and socioeconomic factors were probed by the geodetector.

Based on the literature (Zhao et al. 2019; Tang 2015) and combined with the study area, the average elevation (*X*_1_) and average slope (*X*_2_) of the districts and counties were selected as the two natural geography factors. GDP per capita (*X*_3_), secondary industry value added (*X*_4_), tertiary industry value added(*X*_5_), residential electricity consumption (*X*_6_), total power of agricultural machinery (*X*_7_), the total output value of agriculture, forestry, animal husbandry, and fishery (*X*_8_), sulfur dioxide emission from industrial exhaust (*X*_9_), crop sown area (*X*_10_), disposable income of residents (*X*_11_), industrial wastewater emission (*X*_12_), population density (*X*_13_), and tourism income (*X*_14_), a total of 12 socioeconomic factors, were selected as the independent variables. The production, living, and ecological space area in different periods in Qilian Mountains was used as the dependent variable; the natural geography and socioeconomic factors were detected. The resultant *q*-values characterize the magnitude of a factor’s explanatory power to spatial differentiation factor, and *p*-values less than 0.05.

**Fig. 9:**
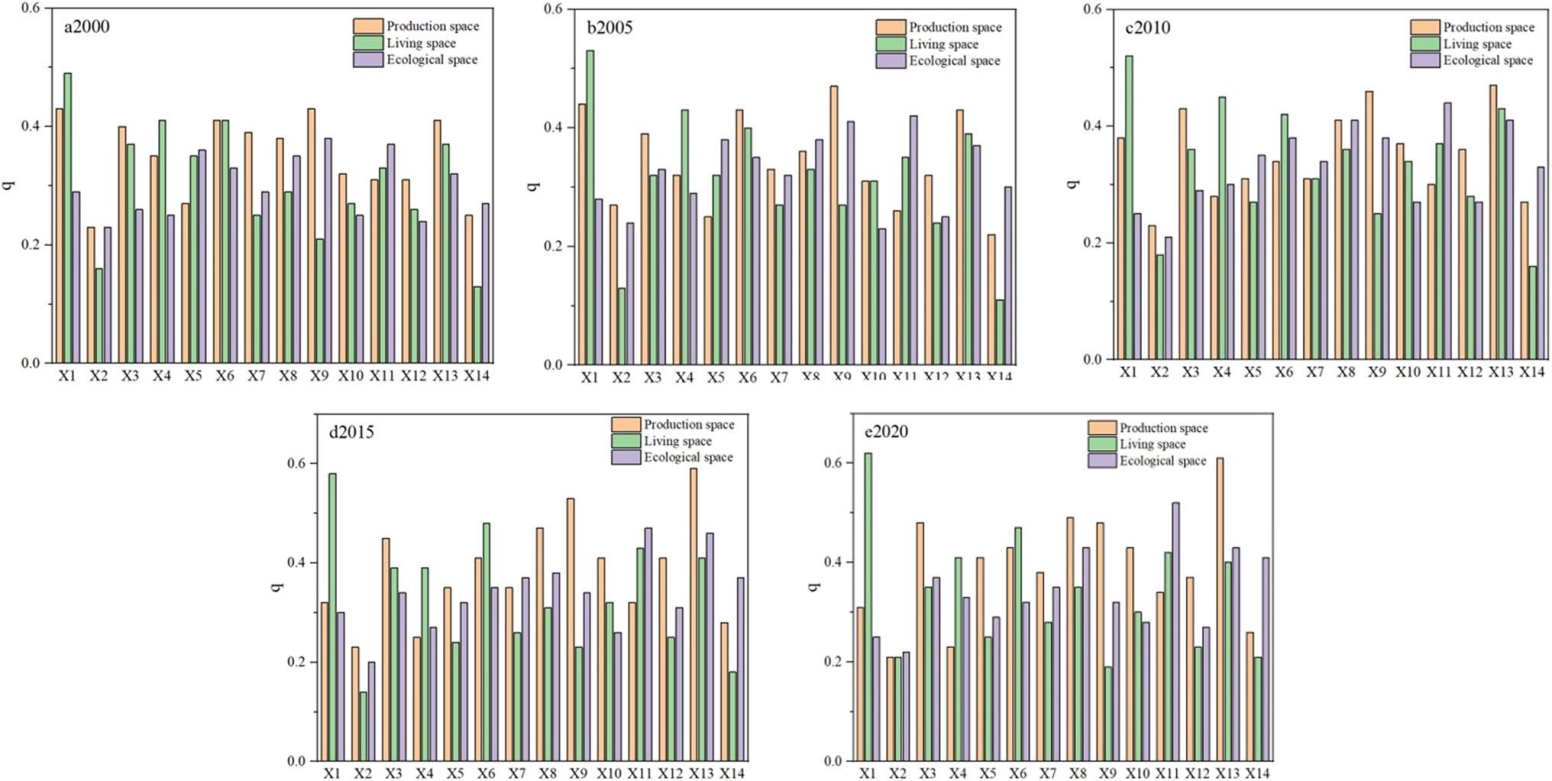
Intensity of the effect of each factor in different periods

The analysis of Fig. 9 shows that the average altitude has a strong influence on living space. The influencing factors on living space and ecological space were quite different, and the slope has a stable effect on production, living, and ecological space. GDP per capita, residential electricity consumption, the total output value of agriculture, forestry, animal husbandry, and fishery, sulfur dioxide emission from the industrial exhaust, and population density were the main drivers of the evolution of production spatial patterns. From 2000 to 2020, the interpretive power of GDP per capita and population density gradually increased, while the interpretive power of the added value of secondary industry decreased year by year. GDP per capita, secondary industry value added, residential electricity consumption, disposable income of residents, and population density were the main drivers of changes in the living space area. The explanatory power of residential electricity consumption and per capita income was increasing, but the value added to the tertiary sector was declining. The disposable income of residents and population density were driven by the evolution of ecological space.

Between 2000 and 2020, the influence of GDP per capita, secondary industry value added, disposable income of residents, population density, and tourism income on the area of ecological space has increased significantly, and the binding effect of tertiary industry value added on the layout of ecological space has decreased. The main drivers of living space and production space were the same, indicating that as the population continued to grow, so did the demand for production and living space. The total output value of agriculture, forestry, animal husbandry, and fishery and the scale of the industry directly determine the area of production and living space and influence the expansion of both. When economic conditions are favorable, the expansion of construction and exploitation of resources has increased ecological pressure.

Qilian Mountains is an extremely important ecological function area of water conservation and a natural barrier to ecological security in western China. It is particularly important to strengthen the ecological space protection in Qilian Mountains, coordinate local green and high-quality development, and pay attention to the transformation and improvement of the spatial distribution of the land.

#### Interaction analysis of impact factors

**Fig. 10:**
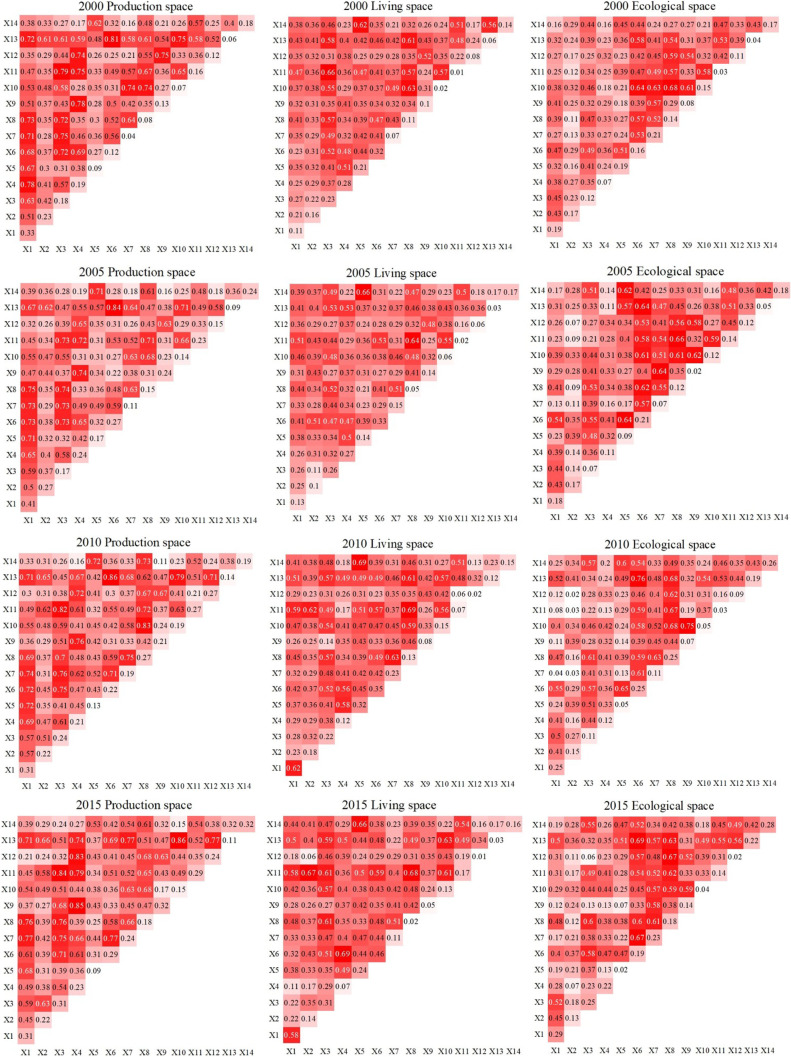

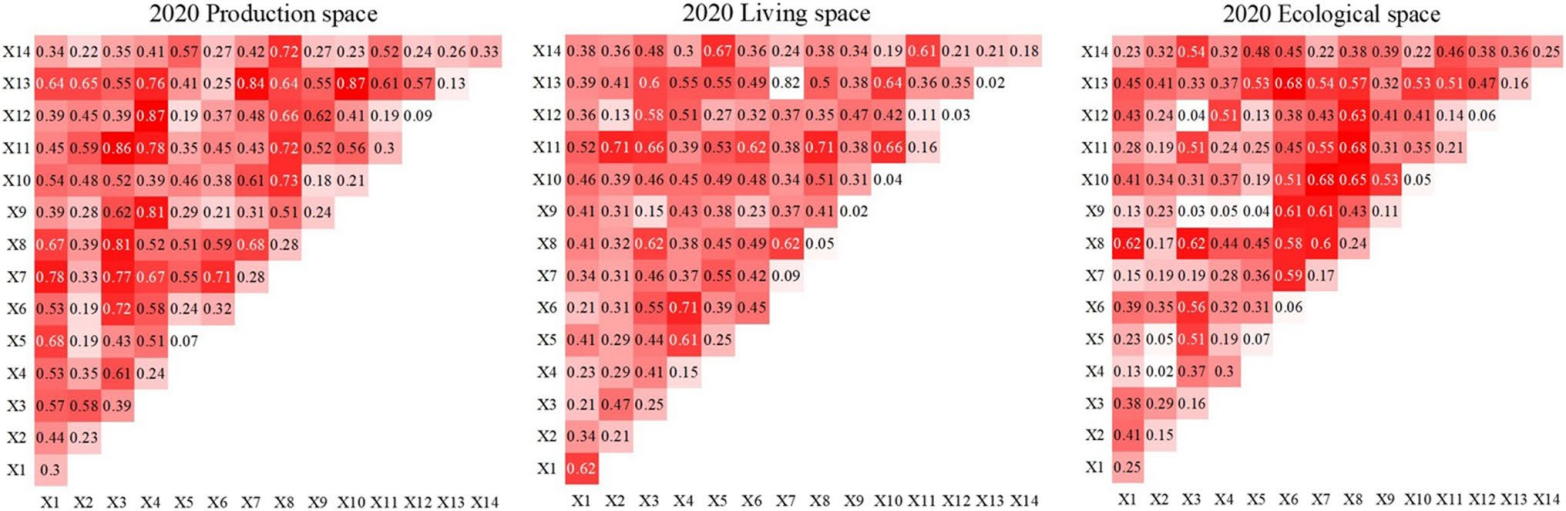
Results of interaction detection

Figure 10 shows the results of the interaction detection analysis using 14 types of impact factors. The interaction between any two factors is either bidirectional or nonlinear, and there was no independent or mutually debilitating relationship. The interaction between any two factors has a greater effect on the production, living, and ecological space than a single factor. The evolution of the production, living, and ecological space in the Qilian Mountains area was influenced by a combination of factors. The higher the q value of the interaction, the greater the influence of two reciprocal counterpart factors on a certain type of production, living, and ecological space.

Between 2000 and 2010, the factors with the strongest impact on production space were residential domestic electricity consumption ∩ population density (0.81, 0.84, 0.86), and between 2010 and 2020, the factors with the strongest impact on production space were secondary industry value added ∩ residential domestic electricity consumption (0.69), crop sown area ∩ population density (0.87), and secondary industry value added ∩ industrial wastewater emissions (0.87). Over the entire 20-year period, the interaction of population density with all other factors was higher, which was related to the stronger univariate explanatory power of population density on production space. Increased population density led to an increase in space demand for agriculture and industry, which has the highest explanatory power for production space.

The analysis of living space showed that the factors with the strongest interaction effects were GDP per capita ∩ disposable income of residents (0.66), tertiary industry value added ∩ tourism income (0.66), the total output value of agriculture, forestry, animal husbandry, and fishery ∩ disposable income of residents (0.69), secondary industry value added ∩ electricity consumption of residents (0.69), and total agricultural machinery power ∩ population density (0.82). Over time, the overall *q* value of the interaction gradually increases, and the interaction of factors affecting the living space increases year by year. The impact of tourism income, the total output value of agriculture, forestry, animal husbandry, and fishery, and secondary industry value added to have more influence on production space than other interaction factors.

The factors that interacted most strongly with ecological space were the total output value of agriculture, forestry, animal husbandry, and fishery ∩ crop sown area (0.68), the total output value of agriculture, forestry, animal husbandry, and fishery ∩ resident disposable income (0.66), resident domestic electricity consumption ∩ population density (0.76), resident domestic electricity consumption ∩ population density (0.69), total agricultural machinery power ∩ crop sown area (0.68), resident domestic electricity consumption ∩ population density (0.68), and the total output value of agriculture, forestry, animal husbandry, and fishery ∩ disposable income of residents (0.68). Factors such as increasing population density, expansion of agricultural land, and increasing disposable income of the population have increased their restrictive effect on ecological space. It verified that anthropogenic disturbance was the dominant influence on ecological, spatial, and functional differentiation.

## Discussion and conclusion

### Discussion

The production and living function index of the Qilian Mountains Nature Reserve generally showed an increasing trend, and the ecological function index decreased during the study period. This indicates that the study area has not reconciled the relationship between economic development and the ecological environment. Previous studies (Liu et al. 2022; Wu et al. 2021) have shown that increasing economic levels at the expense of resources and the environment is not the best path to development. It is recommended that the local government should adjust its development strategy, establish an ecological barrier, and promote the harmonious development of humans and nature.

The distribution of living and production functions in the study area is highly overlapping, the topography is complex, and the backward level of infrastructure and services limits the development of low-value areas. This result is consistent with the findings of Zhang et al. (2022) and Yang et al. (2020b). The government should carry out ecological relocation of residents in the low-value areas and reasonably plan the transfer of residents to towns. In addition, it is necessary to improve rural public services in low-value areas in many aspects, such as education, medical care, and social security.

The Qilian Mountain Nature Reserve is mainly an ecological space; the area of production and living space increased, and ecological space decreased during the study period. This is similar to the research results of Tian et al. (2020). It shows that in the process of urbanization, human activities keep encroaching on forests and grassland, reducing ecological land. Residents should pay attention to ecological, environmental protection and strictly prohibit deforestation. The government should vigorously guide farmers to rely on natural conditions and develop industries such as Chinese herbal medicine cultivation, forestry, and fruit industry, and secondary processing of agricultural products to increase their blood-making capacity, reduce their dependence on natural resources, and promote sustainable development of the regional economy and environment.

It was found that elevation had a significant effect on the evolution of living space, and slope did not have a significant effect on living space. This is inconsistent with the findings of Zhou et al. (2020b). This may be because the slope is the result of a combination of social and natural environmental factors. The large altitude drop in the Qilian Mountains Nature Reserve greatly affects the daily life of the residents. The local government should carry out orderly ecological relocation of villagers in high mountainous areas, evacuate people from the mountains, and reasonably plan the transfer of villagers from protected areas to towns and areas of suitable altitude. Population density is the main socioeconomic factor affecting the change of production space and ecological space area. This is similar to the findings of Wang (2023). Residents should plan their production and living areas rationally, develop special agriculture and plant cash crops, and increase efforts to protect the ecological impact of urban expansion.

### Conclusion

The functional and structural evolution of land reserves reflects the interaction between “human” and “land.” To analyze the evolution characteristics of the function and structure of the production, living, and ecological space in the Qilian Mountains Nature Reserve, this paper constructs an evaluation index system based on the entropy value method. It then measures production-living-ecological functions in 13 districts and counties in the study area, explores the land structure transformation with the help of ArcGIS, estimates the nuclear probability density of the transformation of the production-living-ecological functions, and studies the influential factors by using a geodetector. The study concluded as follows.

During the study period, the production and living function indices in the Qilian Mountains area showed an upward trend. The ecological function indices increased, decreased, then increased again. The functional spatial differences of the production-living-ecological functions were more obvious. The production function area in the north was more developed than it was in the east and south. The area was distributed in the flattest and most convenient area, and the less advantaged area was distributed in the Qilian Mountains’ hilly areas. The distribution of living and production functions was highly overlapping, the topography was complex, and the weak infrastructure and services limited the development of low-value areas. The high-value ecological function areas were distributed in the intact ecological landscape such as Mengyuan and Qilian. Under the influence of economic production and construction, the overall ecological service function value of the study area weakened in 2010–2015. In 2020, the problem of ecological services was improved with the introduction of conservation policies.

During the study period, ecological space dominated the Qilian Mountains area, and the area of production and living space in the Qilian Mountains area continued to increase. The area of production space increased by 858.5 km^2^, mainly from ecological space and unused land. A total of 1004.49 km^2^ of ecological space in northern Qilian and central Menyuan has been transformed into unused land, causing ecological degradation. The central part of Sunan and Qilian has 1147.43 km^2^ of unused land transformed into ecological space, which provides a reference for ecological degradation.

During the study period, the area of production space transferred to the Qilian Mountains area showed obvious spatial clustering characteristics, and the kernel density values transferred were significantly larger than those transferred out. The spatial agglomeration degree of production space reduction was low. The degree of spatial agglomeration of ecological space reduction was high. The reduction of ecological space was the most significant feature of the evolution of the production, living, and ecological space in the Qilian Mountains nature reserve.

During the study period, GDP per capita, the total output value of agriculture, forestry, animal husbandry, and fishery, sulfur dioxide emissions from industrial emissions, and population density were the main factors affecting the production space area. Altitude, residential electricity consumption, and disposable income of residents were the main driving factors affecting the change of living space area. The disposable income of residents, population density, and tourism income are strong constraints on ecological space. The interaction detector revealed that the interaction between any two factors had a greater impact on the production, living, and ecological space in the Qilian Mountains area than any individual factor, indicating that the evolution of the production, living, and ecological space was influenced by a combination of factors.

## Suggestion

The following suggestions will lay the basis for spatial control and management, ecological protection of nature reserves, and green quality development in the Qilian Mountains area.

The medium and high-value areas of production functions, such as Liangzhou, Shandan, and Minle, are more influenced by human activities and economic growth. It is important to consider each region’s environmental and ecological resource-carrying capacity and socioeconomic development, scientifically plan the boundaries of production space, rationally exploit and utilize unused land, improve the efficiency of land use in production space, strengthen border control, and ensure that regional economic development does not come at the expense of opening up ecological space.

The government of the low-value production function area should control the encroachment of living land on farmland and reduce the occupation of production space. Residents need to strengthen the construction of farmland, improve the scale of agricultural production, and achieve a steady increase in agricultural production capacity. In addition, residents can take the ecotourism industry as the lead and entry point to develop a new model of rural tourism and leisure agriculture based on protecting the ecological environment.

For areas with high value of living functions, such as Liangzhou, Ganzhou, and Gaotai, the medium and long-term planning strategy of the national space should be done under the guidance of centralized utilization, conserving living space, focusing on the transformation and improvement of living space functions, and promoting the development of living space. The low-value area of living function should be guided by the government and follow the principle of reasonable planning to carry out village transformation and improvement work. Villages in areas with lagging infrastructure and public services should be relocated to improve the living environment and upgrade the living space.

For medium and high-value ecological function areas such as Menyuan and Qilian, every effort should be made to prioritize ecological space. Human activities and ecological protection can be balanced by limiting human socioeconomic activities in the ecological space. Local authorities should focus on the construction of the nature reserve system while carrying out ecotourism and other green projects, continuously increasing ecological protection and steadily improving the ecological environment capacity and the supply of ecological products.

Low-value ecological function areas should be implemented to return farmland to forestry measures. The local government should speed up the ecological migration process, carry out ecological relocation work in an orderly manner, withdraw people from the mountains, and reasonably plan the transfer of villagers from protected areas to towns. In addition, local environmental protection departments need to carry out pollution surveys and implement treatment projects for polluted environments to ensure ecological safety.

## Research limitations and prospects

Because the data is difficult to obtain, only district and county-level indicators are selected. The impact factors also have shortcomings. Subsequent studies can consider indicators such as policy systems, legal regulations, and other indicators, improve the selection of indicators, and, if conditions permit, fully explore the factors affecting the development of production, living, and ecological space. In addition, future research should include spatial analysis, econometric modeling, and other methods to further study the mechanism of production, living, and ecological spaces from multiple perspectives and put forward targeted policy recommendations.

## Data Availability

The data that support the findings of this research are available from the corresponding author upon reasonable request.

## References

[CR1] Bennett EM, Peterson GD, Gordon LJ (2009). Understanding relationships among multiple ecosystem services. Ecol Lett.

[CR2] Brown G, Raymond CM (2014). Methods for identifying land use conflict potential using participatory mapping. Landsc Urban Plan.

[CR3] Chen WX, Zeng J, Zhong MX, Pan SP (2021). Coupling analysis of ecosystem services value and economic development in the Yangtze River economic belt: a case study in Hunan Province, China. Remote Sensing.

[CR4] Chen C, Jin ZF (2015). Evolution of rural settlement land use pattern in developed areas: a case study of Huishan District, Wuxi City. Geographical Research.

[CR5] Chen WX, Li JF, Zeng J (2019). Spatial heterogeneity and formation mechanism of eco-environmental effects of land use change In China. Geogr Res.

[CR6] Deng YX, Yang R (2021). Influence mechanism of production-living-ecological space changes in the urbanization process of Guangdong Province, China. Land.

[CR7] François-Rémi M, Pierre-Yves L (2022). Towards a generic theoretical framework for pattern-based LUCC modeling: an accurate and powerful calibration–estimation method based on kernel density estimation. Environ Model Softw.

[CR8] Gao SQ, Yang L, Jiao HZ (2022) Changes in and patterns of the tradeoffs and synergies of production-living-ecological space: a case study of Longli County, Guizhou Province, China. Sustainability 14(14)

[CR9] Geng S, Zhu W, Shi P (2019). A functional land use classification for ecological, production and living spaces in the Taihang Mountains. J Resour Ecol.

[CR10] Gong J, Jin TT, Cao EJ (2022). Is ecological vulnerability assessment based on the VSD model and AHP-entropy method useful for loessial forest landscape protection and adaptative management? A case study of Ziwuling Mountain Region, China. Ecol Indic.

[CR11] Han Z, Meng J, Zhu L, Cheng H, Wu Y, Wei C (2022) Quantifying trade-offs of land multifunctionality evaluated by set pair analysis in ecologically vulnerable areas of northwestern China. Land Degrad Dev 33(12)

[CR12] Lu S, Zhou M, Guan X, Tao L (2015). An integrated GIS-based interval-probabilistic programming model for land-use planning management under uncertainty – a case study at Suzhou, China. Environ Sci Pollut Res.

[CR13] Liu CF, Wang YX, He RD (2019). An analysis framework for the identification and optimization of habitat space based on residents’ behavior. J Nat Resour.

[CR14] Lin G, Jiang D, Fu J, Cao C, Zhang D (2020). Spatial conflict of production-living-ecological space and sustainable-development scenario simulation in Yangtze River delta agglomerations. Sustainability.

[CR15] Li YJ, Zhang Q, Wang LZ (2020). Regional environmental efficiency in China: an empirical analysis based on entropy weight method and non-parametric models. J Clean Prod.

[CR16] Luo GP, Zhou CH, Chen X, Li Y (2008). A methodology of characterizing status and trend of land changes in oases: a case study of Sangong River watershed, Xinjiang, China. J Environ Manag.

[CR17] Li YR, Cao Z, Long HL, Liu YS, Li WJ (2017). Dynamic analysis of ecological environment combined with land cover and NDVI changes and implications for sustainable urban-rural development: the case of Mu Us Sandy Land, China. J Clean Prod.

[CR18] Liu J, Xu QL, Yi JH, Huang X (2022). Analysis of the heterogeneity of urban expansion landscape patterns and driving factors based on a combined Multi-Order Adjacency Index and Geodetector model. Ecol Indic.

[CR19] Li X, Lv X, Yin RM, Bin F, Tao J (2021). Spatial equilibrium state and its time evolution of the multi-functionalization of regional land use in the eastern China. Pol J Environ Stud.

[CR20] Li JS, Sun W, Li MY, Meng LL (2021). Coupling coordination degree of production, living and ecological spaces and its influencing factors in the Yellow River Basin. J Clean Prod.

[CR21] Mikelsone E, Atstaja D, Koval V, Uvarova I, Mavlutova I, Kuzmina J (2021) Exploring sustainable urban transformation concepts for economic development. Stud Appl Econ 39(5)

[CR22] Pan Y, Zhu J, Zhang YJ, Li ZN, Wu JX (2022). Poverty eradication and ecological resource security in development of the Tibetan Plateau. Resour Conserv Recycl.

[CR23] Qin F, Fukamachi K, Shibata S (2022). Land-use/landscape pattern changes and related environmental driving forces in a Dong Ethnic Minority Village in Southwestern China. Land.

[CR24] Tao J, Lu Y, Ge D, Dong P, Gong X, Ma X (2022) The spatial pattern of agricultural ecosystem services from the production-living-ecology perspective: a case study of the Huaihai Economic Zone, China. Land Use Policy 122(9)

[CR25] Tang Z (2015). An integrated approach to evaluating the coupling coordination between tourism and the environment. Tour Manage.

[CR26] Tian FH, Li MY, Han XL, Liu H, Mo BX (2020). A production–living–ecological space model for land-use optimisation: a case study of the core Tumen River region in China. Ecol Model.

[CR27] Wang J, Lin YF, Xu YQ (2018). Land-use changes and land policies evolution in China’s urbanization processes. Land Use Policy.

[CR28] Wang H (2021). The impact of shale oil and gas development on rangelands in the Permian basin region: an assessment using high-resolution remote sensing data. Remote Sens.

[CR29] Wang Y (2022). Development characteristics, influencing mechanism and coping strategies of resource-based cities in developing countries: a case study of urban agglomeration in Northeast China. Environ Sci Pollut Res.

[CR30] Wang Y, Yuan G, Yan Y, Zhang X (2020). Evaluation of sustainable urban development under environmental constraints: a case study of Jiangsu Province China. Sustainability.

[CR31] Wang T, Kazak J, Han Q, de Vries B (2019). A framework for path-dependent industrial land transition analysis using vector data. Eur Plan Stud.

[CR32] Wang JF, Xu CD (2017). Geodetectors: principles and prospects. J Geogr.

[CR33] Wang Y (2023). Spatial–temporal evolution of “Production-Living-Ecologica” function and layout optimization Strategy in China: a case study of Liaoning Province, China. Environ Pollut Res.

[CR34] Wei L, Zhou L, Sun DQ (2022). Evaluating the impact of urban expansion on the habitat quality and constructing ecological security patterns: a case study of Jiziwan in the Yellow River Basin, China. Ecol Indic.

[CR35] Wei LY, Zhang YJ, Wang LZ, Mi XY, Wu XY, Cheng ZL (2021). Spatiotemporal evolution patterns of “production-living-ecological” spaces and the coordination level and optimization of the functions in Jilin Province. Sustainability.

[CR36] Wei C, Lin QW, Yu L, Zhang HW, Ye S, Zhang D (2021). Research on sustainable land use based on production–living–ecological function: a case study of Hubei Province, China. Sustainability.

[CR37] Wu JS, Wang ZDN, H, Li XC,  (2021). What is the future for production-living-ecological spaces in the Greater Bay Area? A multi-scenario perspective based on DEE. Ecol Indic.

[CR38] Xie GD, Zhen L, Zhang CX (2010). Assessing the multifunctionalities of land use in China. J Resour Ecol.

[CR39] Xi JC, Wang XG, Kong QQ (2014). Evolution of rural settlements and land use patterns in tourist areas: a case study of three tourist villages in the Nosanpo tourist area. J Geogr.

[CR40] Xie X, Li X, Fan H (2021). Spatial analysis of production-living-ecological functions and zoning method under symbiosis theory of Henan, China. Environ Sci Pollut Res.

[CR41] Xiao XY, Huang X, Jiang LL, Jin CX (2022). Empirical study on comparative analysis of dynamic degree differences of land use based on the optimization model. Geocarto Int.

[CR42] Yao ZH, Wang B, Huang J (2021) Analysis of land use changes and driving forces in the Yanhe River Basin from 1980 to 2015. J Sensors 6692333

[CR43] Yang YY, Bao WK, Liu YS (2020). Coupling coordination analysis of rural production-living-ecological space in the Beijing-Tianjin-Hebei region. Ecol Ind.

[CR44] Yang Y, Bao W, Li Y, Wang Y, Chen Z (2020). Land use transition and its eco-environmental effects in the Beijing–Tianjin–Hebei Urban Agglomeration: a production–living–ecological perspective. Land.

[CR45] Zhang YN, Long HL, Tu SS, Ge DZ (2019). Spatial identification of land use functions and their tradeoffs synergies in China: implications for sustainable land management. Ecol Ind.

[CR46] Zhang SL, Guan ZL, Liu Y, Zheng FM (2022). Land use/cover change and its relationship with regional development in Xixian New Area, China. Sustainability.

[CR47] Zhou L, Zhou C, Che L (2020). Spatio-temporal evolution and influencing factors of urban green development efficiency in China. J Geog Sci.

[CR48] Zhou L, Dang XW, Mu HW, Wang B, Wang SH (2020). Cities are going uphill: slope gradient analysis of urban expansion and its driving factors in China. Sci Total Environ.

[CR49] Zhao X, Calvin KV, Wise MA (2020). The critical role of conversion cost and comparative advantage in modeling agricultural land use change. Clim Chang Econ.

[CR50] Zhao HB, Wei JC, Sun DQ, Wang S, Liu YX, Tan JT (2021). Multiscale coupling coordination of production-life-ecological space within large cities: the case of Zhengzhou City. Resour Sci.

[CR51] Zhao YQ, Cheng JH, Zhu YG, Zhao YP (2021). Spatiotemporal evolution and regional differences in the production-living-ecological space of the urban agglomeration in the middle reaches of the Yangtze River. Environ Res Public Health.

[CR52] Zhao J, Zhao YL (2023). Synergy/trade-offs and differential optimization of production, living, and ecological functions in the Yangtze River economic Belt, China. Ecol Indic.

[CR53] Zhao X, Tang F, Zhang PT (2019). Dynamic simulation and characteristic analysis of county “Production-Living-Ecological” spatial conflicts based on CLUE-S model. Acta Ecological Sinica.

[CR54] Zou LL, Wang JY, Hu XD (2018). Theoretical construction and empirical analysis of the classification system of “Production-Living-Ecological Space” at county level in China. China Land Sci.

